# Reproducibility and intratumoral heterogeneity of the PAM50 breast cancer assay

**DOI:** 10.1007/s10549-023-06888-1

**Published:** 2023-03-09

**Authors:** Amber N. Hurson, Alina M. Hamilton, Linnea T. Olsson, Erin L. Kirk, Mark E. Sherman, Benjamin C. Calhoun, Joseph Geradts, Melissa A. Troester

**Affiliations:** 1 Department of Epidemiology, Gillings School of Global Public Health, University of North Carolina at Chapel Hill, Chapel Hill, NC USA; 2 Division of Cancer Epidemiology and Genetics, National Cancer Institute, National Institutes of Health, Rockville, MD USA; 3 Department of Pathology and Laboratory Medicine, University of North Carolina at Chapel Hill, Chapel Hill, NC USA; 4 Quantitative Health Sciences Research, Mayo Clinic, Jacksonville, FL USA; 5 Department of Pathology and Laboratory Medicine, East Carolina University Brody School of Medicine, Greenville, NC USA; 6 Department of Epidemiology, University of North Carolina at Chapel Hill, 253 Rosenau, CB# 7435, Chapel Hill, NC 27599 USA

**Keywords:** Intratumoral heterogeneity, Discordance, PAM50, Gene signature

## Abstract

**Background:**

The PAM50 assay is used routinely in clinical practice to determine breast cancer prognosis and management; however, research assessing how technical variation and intratumoral heterogeneity contribute to misclassification and reproducibility of these tests is limited.

**Methods:**

We evaluated the impact of intratumoral heterogeneity on the reproducibility of results for the PAM50 assay by testing RNA extracted from formalin-fixed paraffin embedded breast cancer blocks sampled at distinct spatial locations. Samples were classified according to intrinsic subtype (Luminal A, Luminal B, HER2-enriched, Basal-like, or Normal-like) and risk of recurrence with proliferation score (ROR-P, high, medium, or low). Intratumoral heterogeneity and technical reproducibility (replicate assays on the same RNA) were assessed as percent categorical agreement between paired intratumoral and replicate samples. Euclidean distances between samples, calculated across the PAM50 genes and the ROR-P score, were compared for concordant vs. discordant samples.

**Results:**

Technical replicates (*N* = 144) achieved 93% agreement for ROR-P group and 90% agreement on PAM50 subtype. For spatially distinct biological replicates (*N* = 40 intratumoral replicates), agreement was lower (81% for ROR-P and 76% for PAM50 subtype). The Euclidean distances between discordant technical replicates were bimodal, with discordant samples showing higher Euclidian distance and biologic heterogeneity.

**Conclusion:**

The PAM50 assay achieved very high technical reproducibility for breast cancer subtyping and ROR-P, but intratumoral heterogeneity is revealed by the assay in a small proportion of cases.

## Introduction

Breast cancer is a heterogenous disease that includes subtypes with distinct biology, clinical characteristics, and prognosis. Thus, multigene assays for breast cancer subtype classification are used in research and treatment. Generally, these assays are run on a single sample from a representative tissue block. When assessing the gene expression patterns of a sampled specimen for diagnostic and treatment purposes, the aim is to sample a part of the tumor that best reflects tumor biology and predicts prognosis and treatment response. However, studies have demonstrated that spatial heterogeneity of biomarker expression, as well as chromosomal and genomic alterations, are common features of breast tumors. Morphologically distinct tumor regions may have distinct genetic aberrations [1, 2].

Considering standard clinical markers, heterogeneity of ER expression has been shown to be relatively low [3, 4], while heterogeneity of expression of HER2, PR, and Ki67 is known to be high [3–7]. For example, up to 15% of breast tumors show discordance of HER2 status by FISH across randomly sampled sections, with heterogeneity being most frequent (up to 27%) in tumors with an equivocal (2 +) HER2 score [8]. Further, a recent study found Ki67 heterogeneity present in 18% of sampled breast tumors, which included a high frequency of two types of heterogeneity: as a gradient of increasing staining towards the tumor edge (likely representing a common technical artifact) and as hot spots [9]. Beyond the clinical uncertainty associated with biomarker discordance, it is believed that intratumoral heterogeneity may have prognostic relevance, with high heterogeneity breast tumors associated with worse survival [10, 11].

In light of this spatial heterogeneity, multigene assays have been viewed as a more stable solution to breast tumor subtyping. In particular, the PAM50 assay was designed to identify five intrinsic subtypes (Luminal A, Luminal B, HER2-enriched, Basal-like, and Normal-like), which have both etiologic and prognostic significance. This assay and others (e.g., Oncotype DX and Mammaprint) are recommended clinically to guide treatment decisions [12–15], highlighting the importance of understanding the potential for outcome misclassification in studies of breast cancer subtype. Outcome misclassification could arise from technical or biological heterogeneity. To estimate the upper bound on the frequency of technical and biological variability in PAM50 classification, we selected morphologically heterogenous and/or spatially separate breast tumor regions and performed technical replication studies across formalin-fixed paraffin-embedded samples of breast tissue.

## Methods

### Study population

The UNC Normal Breast Study (NBS), described previously [16], was a hospital-based study conducted at UNC Hospitals in Chapel Hill, North Carolina from 2009 to 2013. The study recruited women undergoing breast surgery and samples of grossly normal-appearing breast tissue and tumor tissue were extracted, then snap frozen or paraffin embedded. The present study is of tumor tissue only.

### Tumor samples

Tumors from 49 patients were assayed repeatedly (2–3 times) at different spatial locations (central versus peripheral tumor), specifically targeting regions that had distinct histological characteristics (e.g., tumor cellularity, tumor grade, admixture of DCIS or benign epithelium, and inflammatory response). From the formalin-fixed paraffin-embedded (FFPE) tumor block, a top slide was cut and stained with hematoxylin & eosin. Using this slide as a guide, up to three 1-mm cores were cut from histologically distinct regions of each tumor block. RNA was isolated from each core using the RNeasy FFPE kit (cat# 73504) from Qiagen and run on a Nanostring codeset containing 417 probes, including 11 housekeeping genes. There were 95 samples analyzed. We excluded 5 samples in technical quality control due to high missingness across genes, which left 90 tumor samples from 48 patients. Samples with no pair were excluded (*n* = 11), resulting in a final analytic sample of 85 tumor samples across 40 patients (35 patients with *n* = 2 samples and 5 patients with *n* = 3 samples). A second pathologist scored the tumor specimens for several histologic characteristics. Mitotic activity was assessed by recording the maximum number of mitoses in a single 20 × field, with heterogeneous cores defined as those that differ by two or more mitotic figures per field. Additionally, presence of immune infiltration (present/absent), distance from the biopsy site (adjacent/away) and location of the core on the tumor (peripheral/central) were assessed for each core. The biopsy site, which is the focus of inflammation and healing induced by a previous core needle biopsy, was present/identifiable for half of the tumors in the analytic sample. Tumors were categorized as concordant or discordant for each histologic characteristic.

To assess technical reproducibility, we generated 1–2 technical replicates of all 90 tumor samples, wherein each RNA sample was run on the assay at least twice. After sample cleaning, six samples were left without a pair and were subsequently excluded, leaving a total of 207 replicate tumor samples across 87 patients (54 patients with *n* = 2 samples and 33 patients with *n* = 3 samples). When estimating technical reproducibility, we also included technical replicates of tumor samples from a second study—Phase 3 of the Carolina Breast Cancer Study (CBCS3)—which is a population-based study that enrolled breast cancer cases occurring in North Carolina during the same study interval (2008–2013). Tumors from 57 patients in CBCS3 were assayed repeatedly at different spatial regions and 1–2 technical replicates were generated for 57 tumors. Throughout this manuscript when we refer to “technical reproducibility” or “technical replicates” we are referring to the repeated samples of the same RNA. When we refer to “intratumoral reproducibility” or “intratumoral samples” we are referring to the separate cores from a patient’s tumor block.

Gene expression was quantified using a research version of the PAM50 assay on the NanoString platform. The expression data were quality checked and cleaned using our previously described pipeline, then normalized as previously described using the remove unwanted variation (RUV) method [17–19]. Samples were classified for intrinsic subtype (Luminal A, Luminal B, HER2-enriched, Basal-like, or Normal-like) and for risk of recurrence (ROR-P) score (continuous) and group (low, medium, or high).

### Statistical analysis

Reproducibility was assessed by calculating percent agreement of intrinsic subtype and ROR-P group across paired intratumoral and replicate samples. Additionally, we calculated the Euclidean distances of the expression levels of the 50 genes (stratified by discordance/concordance of intrinsic subtype) and of the ROR-P score (stratified by discordance/concordance of ROR-P group) within each set of intratumoral and replicate samples. Euclidean distance is a statistical metric commonly used to quantify the similarity between vectors. Wilcoxon rank sum tests were used to test whether the median of the distances differed between concordant and discordant samples.

## Results

**Fig. 1:**
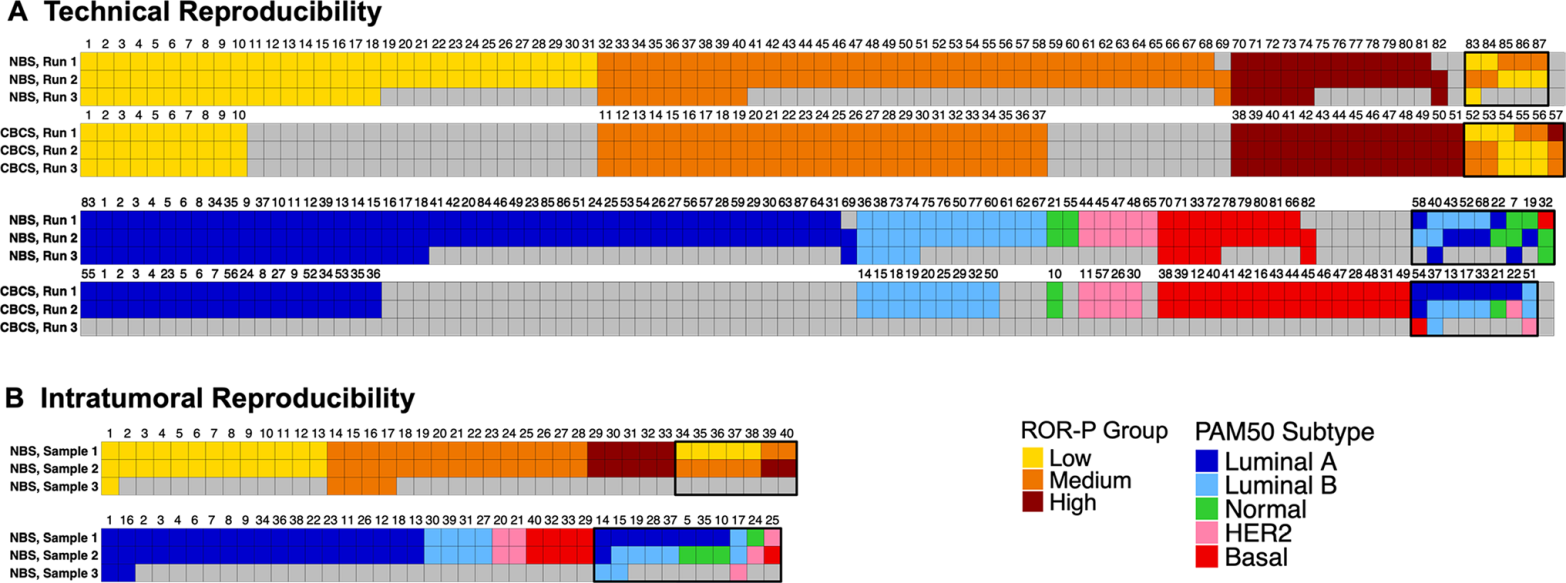
Concordance and percent agreement of PAM50 subtype and ROR-P group between technical replicates (**A**) and between intratumoral samples (**B**). Grey cells indicate unavailable samples. *CBCS* = Carolina Breast Cancer Study, *NBS* = Normal Breast Study, *PAM50* = Prediction Analysis of Microarray 50, *ROR-P* = relapse score based on subtype and proliferation

**Fig. 2:**
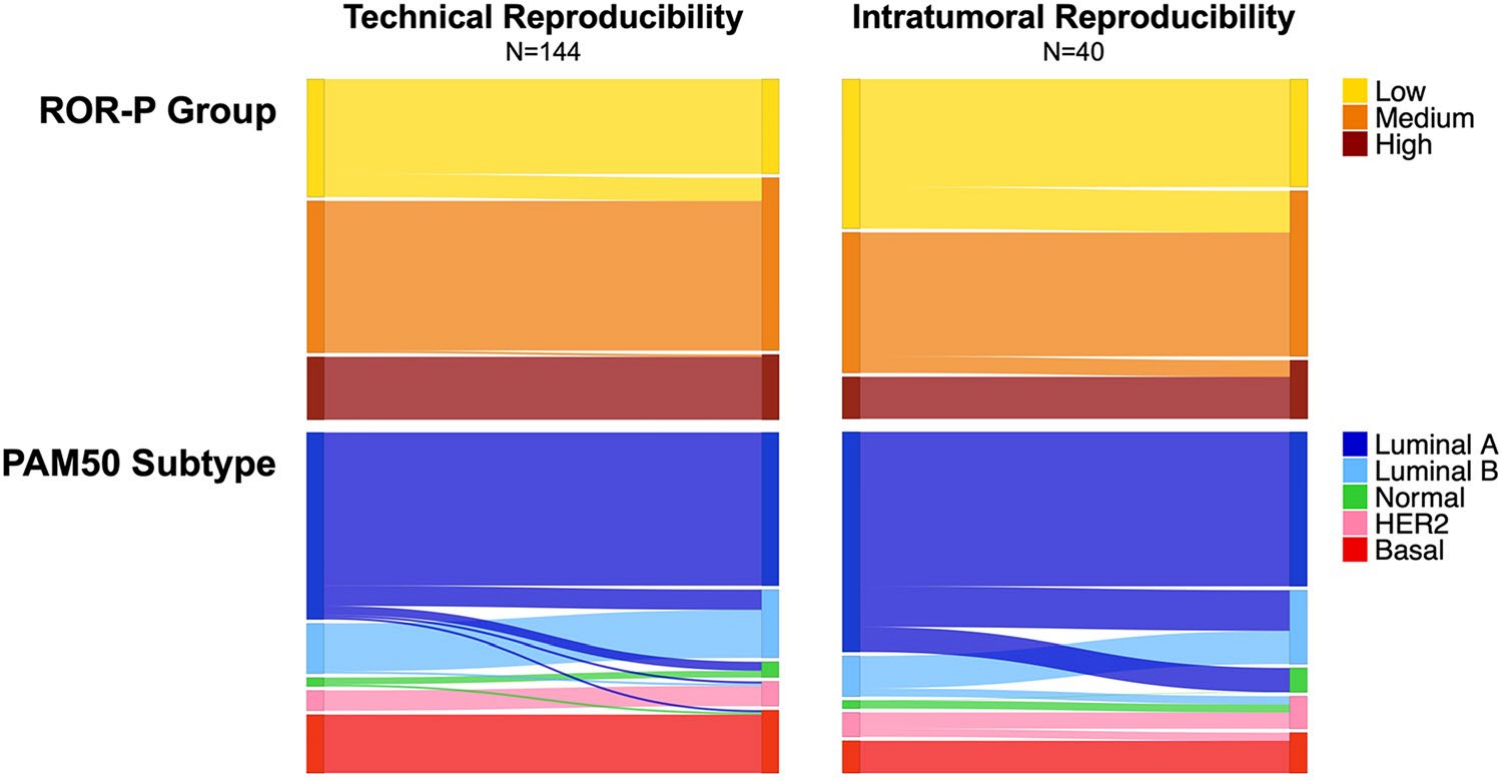
Concordance of ROR-P group and PAM50 subtype between technical replicates and between intratumoral samples. *PAM50* = Prediction Analysis of Microarray 50, *ROR-P* = relapse score based on subtype and proliferation

To assess technical reproducibility of the PAM50 assay, we performed Nanostring analysis in duplicate on the same RNA extract from 144 FFPE tumor samples from two different studies (87 NBS samples and 57 CBCS samples). Technical agreement was very high, but varied according to categorical predictor. For the three-class predictor—ROR-P (high, medium, low)—133 of 144 pairs were concordant for ROR-P group (93% agreement; Figs. 1, 2). All the discordant pairs changed between adjacent ordinal categories (*n* = 10 from low to medium and *n* = 1 from medium to high; Fig. 1). For PAM50 subtype, classification includes a larger number of categories and therefore we expected lower concordant rates. Of 144 pairs, 127 were concordant for PAM50 subtype (90% agreement; Figs. 1, 2). About half of the discordant cases (9 of 17) were Luminal A in one replicate and Luminal B in the second replicate (Fig. 1). Only one sample changed between a Luminal subtype and Basal-like. These technical reproducibility findings provide a comparison point for the intratumoral comparisons.

To assess intratumoral (i.e., biological) reproducibility of the PAM50 assay, we performed a ‘challenge’ study that specifically oversampled histologically distinct regions within each tumor. Of the 40 tumors analyzed, 33 were concordant for ROR-P group (81% agreement) and 29 were concordant for PAM50 subtype (76% agreement; Figs. 1, 2). The pathologist-selected histologically distinct regions were independently scored for a variety of parameters by a second pathologist and 83% showed discordance in at least one histological characteristic. Compared to subtype-concordant samples, paired samples that were discordant for PAM50 subtype had more heterogeneity with respect to the distance of each core from the biopsy site and presence of stromal TILs (Table 1). Heterogeneity of mitotic activity (as recorded by the pathologist) was observed for 57% of samples discordant and 39% of samples concordant for ROR-P group (*n* = 4/7 and *n* = 12/31, respectively; Table 1).

**Table 1:** Tumor and sample characteristics stratified by concordance of PAM50 subtype call and ROR-P group

	PAM50 subtype		ROR-P group	
	Concordant	Discordant	Concordant	Discordant
Technical reproducibility	*N* = 127	*N* = 17	*N* = 133	*N* = 11
High confidence in PAM50 call^a^	118 (93%)	13 (76%)	–	–
Median (range) Euclidean distance, PAM50 genes	3.8 (1.7–20.1)	4.6 (2.5–12.0)	–	–
Median (range) Euclidean distance, ROR-P score	–	–	3.2 (0.2–22.1)	6.6 (1.2–60.8)
Tumor grade				
1/2	73 (58%)	11 (65%)	77 (58%)	7 (64%)
3	53 (42%)	6 (35%)	58 (42%)	4 (36%)
Unknown	1	0	1	0
Tumor size				
≤ 2 cm	55 (43%)	7 (41%)	60 (45%)	2 (18%)
> 2 cm	72 (57%)	10 (59%)	73 (55%)	9 (82%)
Intratumoral reproducibility	*N* = 29	*N* = 11	*N* = 33	*N* = 7
High confidence in PAM50 call^a^	27 (93%)	9 (82%)	–	–
Median (range) Euclidean distance, PAM50 genes	6.7 (3.8–10.8)	7.8 (4.5–12.6)	–	–
Median (range) Euclidean distance, ROR-P score	–	–	8.9 (0.0–28.5)	22.9 (10.7–28.2)
Tumor grade				
1/2	19 (66%)	6 (55%)	21 (64%)	4 (57%)
3	10 (34%)	5 (45%)	12 (36%)	3 (43%)
Tumor size				
≤ 2 cm	13 (45%)	4 (36%)	16 (48%)	1 (14%)
> 2 cm	16 (55%)	7 (64%)	217 (52%)	6 (86%)
Heterogeneity of histology				
Distance from biopsy site	9/16 (56%)	4/4 (100%)	12/18 (67%)	1/2 (50%)
Presence of stromal TILs	5/27 (19%)	4/11 (36%)	7/31 (23%)	2/7 (29%)
Mitotic activity	13/27 (48%)	3/11 (27%)	12/31 (39%)	4/7 (57%)
Location of core in the tumor	19/21 (90%)	5/9 (56%)	20/25 (80%)	4/5 (80%)

*PAM50* Prediction Analysis of Microarray 50, *ROR-P* relapse score based on subtype and proliferation, *TILs* tumor infiltrating lymphocytes

^a^Defined as all samples having ≥ 97% confidence in the subtype call

Multigene classifiers can vary in their strength of association with the single sample predictor vector, suggesting that not all samples have high concordance with the standard for that class. Confidence scores and other diagnostics that reflect the strength of the classifier correlation can be a useful indicator of sample quality and may also impact both technical and biological reproducibility. To assess whether the strength of the classification was a factor influencing reproducibility, we evaluated technical and biological replicates for confidence scores using the method of Parker et al. [20] Among technical replicates, samples discordant for PAM50 subtype were less likely to have high confidence subtype calls compared to concordant samples (76% compared to 93%; Table 1). Among intratumoral samples, however, concordant and discordant tumors had similar rates of high confidence calls (93% and 82%, respectively; Table 1). For both the technical replicates and intratumoral samples, the majority of discordant subtype calls were Luminal A with Luminal B, or Luminal A with Normal-like (Figs. 1, 2).

To identify possible predictors of technical or biological discordance, we assessed whether tumor characteristics were associated with discordance. For both the technical replicates and intratumoral samples, the distributions of tumor grade and size were not associated with discordance of PAM50 subtype call. However, we observed non-statistically significant differences in tumor size between ROR-P-concordant and discordant tumors, with discordant samples generally arising from larger tumors (Table 1). Among technical replicates, 82% of discordant samples came from tumors larger than 2 cm compared to 55% of concordant samples. Among intratumoral samples, 86% of discordant samples came from tumors larger than 2 cm compared to 52% of concordant samples.

**Fig. 3:**
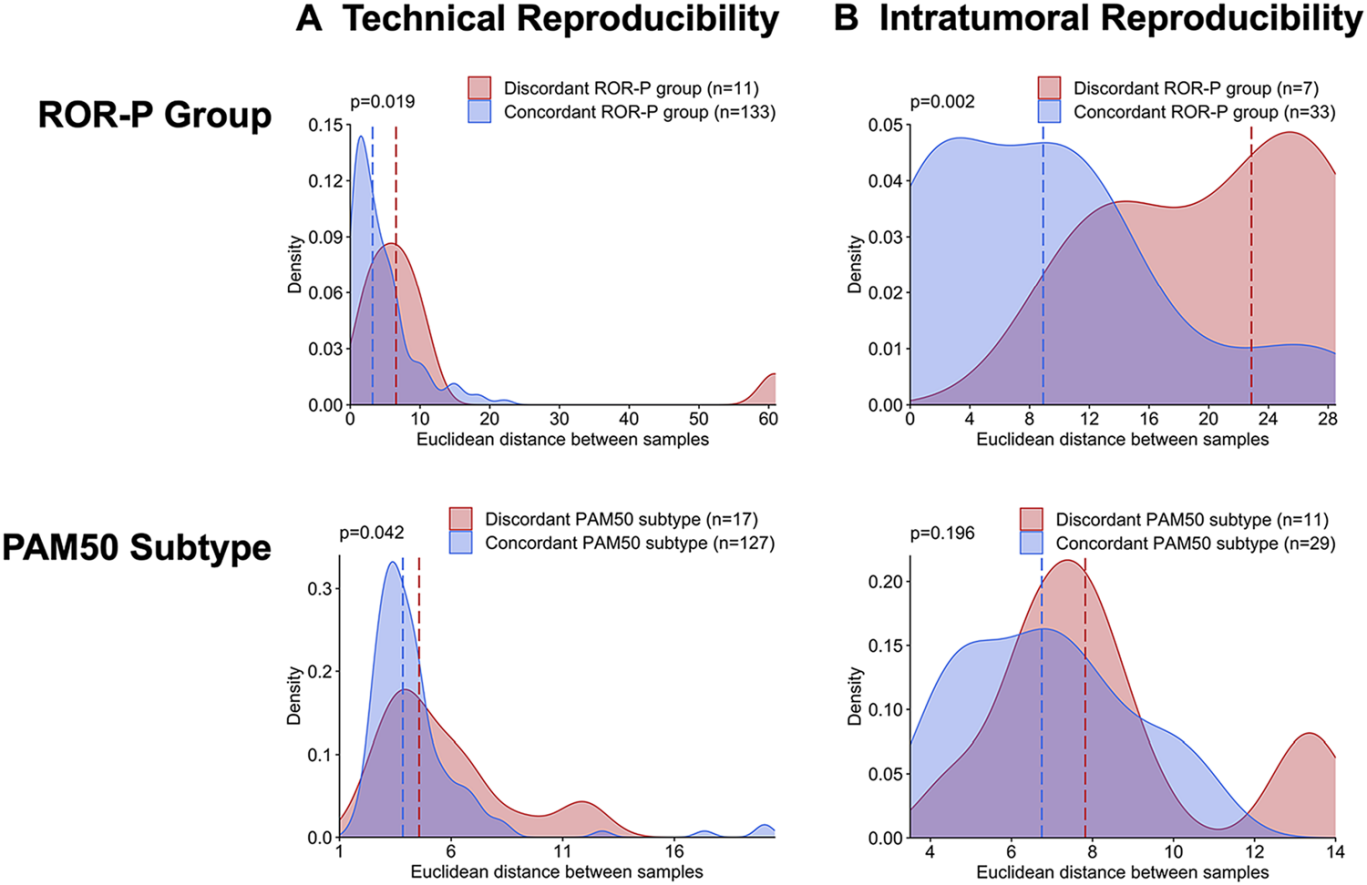
Euclidean distances between the ROR-P scores and expression levels of the 50 genes that comprise the PAM50 classifier for technical replicates (**A**) and intratumoral samples (**B**). *p*-values correspond to the test statistic from a Wilcoxon rank sum test comparing the median of the distances in each group. *PAM50* = Prediction Analysis of Microarray 50, *ROR-P* = relapse score based on subtype and proliferation

While our emphasis was to assess reproducibility of categorical classification, we also evaluated the differences in multigene continuous scores. Specifically, we calculated Euclidean distance between pairs of two or three replicates from each tumor. Figure 3 illustrates the magnitude of the difference in the continuous ROR-P score and in the gene expression levels of the genes used by the PAM50 classifier between paired samples. Among the technical replicates (Fig. 3A), the distances between ROR-P scores and between the gene expression levels were higher for discordant compared to concordant cores, but the distributions were similar and largely overlapping. In contrast, among intratumoral samples (Fig. 3B), the distances between cores were higher for discordant samples—particularly for samples discordant for ROR-P group (*p* = 0.002). The distributions among samples discordant for ROR-P group or PAM50 subtype appear bimodal, with the first peak largely overlapping with the distributions for concordant samples and the second peak corresponding to samples with greater distances among ROR-P scores or among gene expression levels, suggesting that discordant biological replicates had differences beyond expectation for technical variation.

## Discussion

This study examined the extent to which clinically relevant multigene scores for breast cancer are subject to misclassification. Despite observing heterogeneity across replicate samples (from both biological and technical sources), there was moderate-to-strong concordance of PAM50 subtype call and ROR-P groups across technical replicates and spatially distant biological replicates. A slightly higher rate of discordance among samples selected for histologic heterogeneity suggests meaningful variation across tumors, particularly in tumors that are larger size, have higher mitotic variability, and more infiltration of TILs. This biological heterogeneity detected within blocks may be even more pronounced when comparing across separate blocks, suggesting that especially for Luminal A and B tumors, there is potential for outcome misclassification in a proportion (up to 20%) of samples.

We expected to observe some degree of discordance across a tumor, as prior studies have revealed intratumoral genomic heterogeneity of breast tumors. Raychaudhuri et al. assessed heterogeneity in expression levels of several pro-metastatic miRNAs across multiple regions from each tumor and observed considerable intratumoral heterogeneity (coefficient of variation = 40%) [21]. Similarly, substantial heterogeneity of putative driver genes has been observed across spatially separated breast tumor samples [22]. Interestingly, a study of the impact of intratumoral heterogeneity on the performance of microarray-based assays in breast cancer found the amount of intratumoral variance directly correlated with expression level, with lowly expressed genes having higher heterogeneity [23]. A previous paper by Lopez-Knowles et al. found that poor reproducibility could be linked with low confidence [24]. We observed this pattern for technical replicates; however, we could not explain intratumoral reproducibility by confidence scores alone.

Despite previous reports suggesting high genomic intratumoral instability, heterogeneity of multigene scores has been found to be relatively minimal. The robustness of the PAM50 classifier and other multigene classifiers has been attributed to improved filtration of background noise and aggregation across multiple signals, presumably resulting in more accurate classification. For example, the intrinsic gene subset that gave rise to the PAM50 classifier was devised to maximize heterogeneity of expression between tumors and minimize heterogeneity of expression within tumors [25]. High agreement of PAM50 intrinsic subtype classification has been reported, with 11/12 and 4/6 tumors with concordant samples (based on RNA-sequencing and microarray-based gene expression profiling, respectively) [22]. Several studies have also found minimal intratumoral heterogeneity of the recurrence score from the Oncotype DX assay, with the continuous score having high concordance between core biopsies and resections (*n* = 49 patients, Pearson’s *r* = 0.86) [26] and between multiple tumor blocks (*n* = 19 patients, *r* = 0.94) [27]. When categorizing the Oncotype DX recurrence score into low, intermediate, and high categories, three of eight patients had one core in a higher risk category than the other three cores [28]. Low intratumoral heterogeneity has also been reported for the EndoPredict [29] and MammaPrint [30, 31] risk of recurrence scores. Results from these studies of intratumoral heterogeneity of multigene scores are comparable to the present study, where there was 81% and 76% intratumoral agreement of ROR-P group and PAM50 subtype, respectively.

While rare, some discordance in ROR-P group between intratumoral samples was observed (19% disagreement), with large tumor size (> 2 cm) and heterogeneity of mitotic activity between cores observed as potential predictors of ROR-P group discordance. This raises the question of whether tissue from larger tumors should be reviewed for histologic features prior to taking samples for RNA extraction and testing. There is no clear indication in the literature that there is more variation in larger tumors, but it is a reasonable expectation that might be true in some cases. For example, different blocks might yield more heterogeneity of ER expression [32]. This also may depend on patterns for ordering the assays. One paper suggests that multigene assays are ordered more frequently on large tumors [33], but according to Medicare data they seem to be ordered for small tumors if they appear to be high risk [34]. The current evidence may not justify targeted selection of sampling regions because the clinical significance of the potential heterogeneity is unknown. Nevertheless, the use of this technology seems to be growing and future work might consider whether large tumors that produce multiple blocks require multiple assays.

This study has several strengths. First, we conducted a detailed histopathology review of samples to try to maximize the opportunity to detect heterogeneity. Additionally, the estimate of baseline technical variation provides a metric with which to compare the observed discordance to the assay-based discordance. It is also important that although the probes used may differ slightly from those in the commercial version of the assay, we have used the same genes and algorithm as the Prosigna assay. With proper data normalization, this has been shown to be robust across datasets [20]. Any discrepancies relative to actual clinical subtype is expected to be random.

This study also has several limitations. This analysis was designed to challenge intra-block variation (reproducibility within a block) and does not inform on the level of variation across lesions from different tumor blocks. We did not assess whether the level of reproducibility observed in the present study was significantly different than some established threshold. Rather, our goal was to describe the reproducibility in a diverse cohort where we had multiple measures. Additionally, the modest sample size does not allow for stratification by other tumor characteristics (e.g., grade or subtype). Lastly, the goal of this study was to identify the maximum amount of misclassification that may occur, yet a cohort of breast cancer cases with more aggressive tumors may observe higher rates of intratumoral heterogeneity. However, the distribution of tumor characteristics in the present study is similar to those in other population-based studies (e.g., CBCS).

Our findings indicate that intratumoral heterogeneity is not likely to be a major impediment to the interpretation of multigene scores for breast tumors. The PAM50 classifier is relatively robust to repeated sampling and, in most applications, the ROR-P score seems highly reproducible. Achieving the level of discordance observed in this study required us to selectively sample heterogenous appearing tumor regions, therefore representing an upper bound on the level of discordance expected from a single tumor block. While intratumoral heterogeneity is not expected to be a major cause of misclassification when applying multigene signatures, heterogeneity does exist within breast tumors and sampling may cause some misclassification of tumors, with relevance for breast cancer treatment decisions.

## Data Availability

The datasets generated during and/or analyzed during the current study are not publicly available due to some human subjects restrictions, but may be available from the corresponding author on reasonable request.
